# Phase III, randomized, open-label study of durvalumab (MEDI4736) in combination with tremelimumab or durvalumab alone versus platinum-based chemotherapy in first-line treatment of patients with advanced/metastatic NSCLC: MYSTIC

**DOI:** 10.1186/2051-1426-3-S2-P171

**Published:** 2015-11-04

**Authors:** Naiyer Rizvi, Fabrice Barlesi, Julie Brahmer, Enriqueta Felip, Patrick Forde, Marina Garassino, Sarah Goldberg, Johan Vansteenkiste, Anthony Jarkowski, Stuart McIntosh, Luping Zhao, Scott Antonia

**Affiliations:** 1 Columbia University Medical Center, New York, NY, USA; 2 Aix Marseille University-Assistance Publique Hôpitaux de Marseille, Marseille, France; 3 Sidney Kimmel Comprehensive Cancer Center at Johns Hopkins, Baltimore, MD, USA; 4 Vall d'Hebron University Hospital, Barcelona, Spain; 5 Fondazione IRCCS Istituto Nazionale dei Tumori, Milano, Italy; 6 Yale University, Yale Cancer Center, New Haven, CT, USA; 7 University Hospitals KU Leuven, Leuven, Belgium; 8 AstraZeneca, Gaithersburg, MD, USA; 9 AstraZeneca, Macclesfield, United Kingdom; 10 H. Lee Moffitt Cancer Center and Research Institute, Tampa, FL, USA

## Background

Platinum-based doublets are standard of care (SoC) first-line treatment for advanced NSCLC, but durable benefit is observed infrequently, with resistance to chemotherapy invariably developing. The blockade of immune checkpoints is a promising novel approach in cancer treatment. Blocking co-inhibitory molecules, such as programmed cell death-1 (PD-1) and cytotoxic T-lymphocyte-associated antigen-4 (CTLA-4), from binding with their ligands can restore T-cell responses against tumors. As these pathways are non-redundant, dual targeting may have additive or synergistic antitumor activity. Durvalumab (MEDI4736) is a selective, high affinity human IgG1 mAb that blocks programmed cell death ligand-1 (PD-L1) binding to PD-1 (IC_50_ 0.1 nM) and CD80 (IC_50_ 0.04 nM), and tremelimumab is a selective human IgG2 mAb inhibitor of CTLA-4. Durvalumab monotherapy has shown durable responses in a NSCLC cohort from a Phase I/II study in heavily pre-treated patients with solid tumors[1] and durvalumab plus tremelimumab has shown encouraging clinical activity and a manageable safety profile in a Phase Ib study in patients with NSCLC[2]. A comprehensive clinical development program of durvalumab +/- tremelimumab in NSCLC is underway. MYSTIC (NCT02453282) is a global Phase III study to determine the efficacy and safety of durvalumab plus tremelimumab combination therapy or durvalumab monotherapy versus SoC platinum-based doublets in the first-line treatment of patients with advanced or metastatic NSCLC.

## Methods

**Figure 1:**
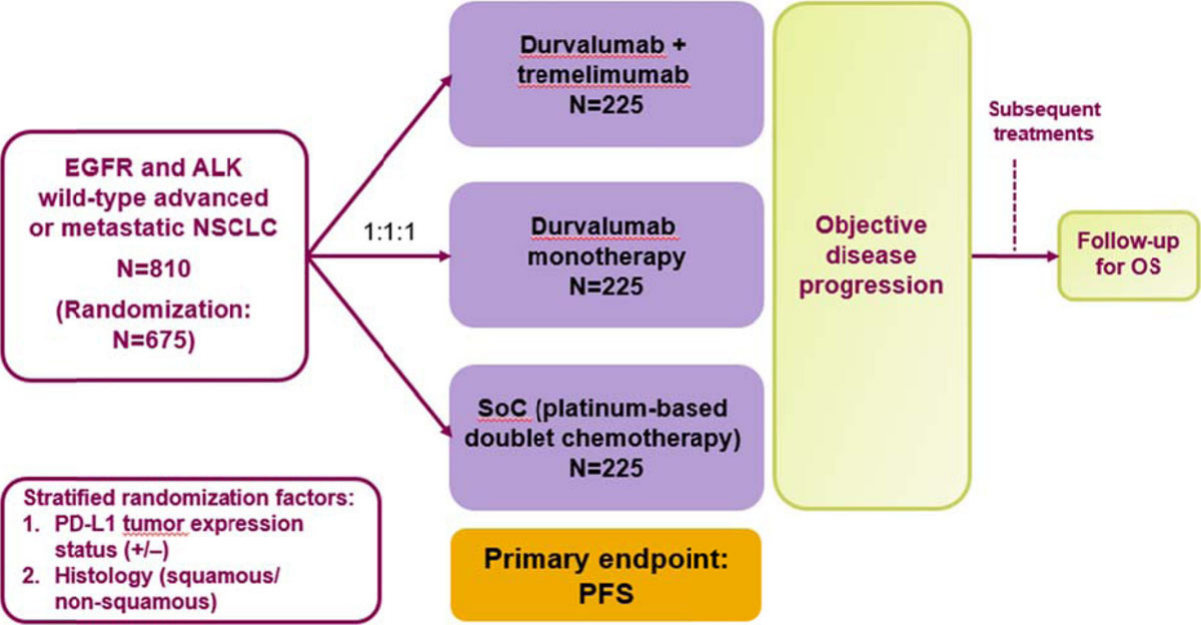


In this randomized, open-label, multicenter, global, Phase III study, 675 immunotherapy- and chemotherapy-naïve patients with advanced or metastatic (Stage IV) NSCLC who are wild-type for epidermal growth factor receptor (EGFR) and anaplastic lymphoma kinase (ALK) will be randomized (1:1:1) to receive durvalumab (20 mg/kg IV every 4 weeks for up to 12 months) plus tremelimumab (1 mg/kg IV every 4 weeks for up to 4 doses); durvalumab monotherapy (20 mg/kg IV every 4 weeks for up to 12 months); or SoC chemotherapy (Figure 1). Stratification factors are PD-L1 status and histology. The primary endpoint is progression-free survival (PFS) using investigator assessments (RECIST v1.1). Secondary endpoints will further assess objective response rate, duration of response, proportion of patients alive and progression free at 12 months, time from randomization to second progression, and overall survival (OS); safety (CTCAE v4.03) and tolerability; health-related quality of life; pharmacokinetics; and immunogenicity. Exploratory outcomes include potential biomarkers of response to treatment assessed on a biopsy at trial entry. Study sites are being opened for recruitment.

## Trial registration

ClinicalTrials.gov identifier NCT02453282.

## References

[B1] Rizvi ASCO 2015 Abstract 8032

[B2] Antonia ASCO 2015 Abstract 3014

